# Minority-group incubators and majority-group reservoirs support the diffusion of climate change adaptations

**DOI:** 10.1098/rstb.2022.0401

**Published:** 2023-11-06

**Authors:** Matthew A. Turner, Alyson L. Singleton, Mallory J. Harris, Ian Harryman, Cesar Augusto Lopez, Ronan Forde Arthur, Caroline Muraida, James Holland Jones

**Affiliations:** Stanford Doerr School of Sustainability, Social Sciences Division, Stanford University, Stanford, CA 94305, USA

**Keywords:** agent-based modelling, homophily, cooperation

## Abstract

Successful climate change adaptation depends on the spread and maintenance of adaptive behaviours. Current theory suggests that the heterogeneity of metapopulation structure can help adaptations diffuse throughout a population. In this paper, we develop an agent-based model of the spread of adaptations in populations with minority–majority metapopulation structure, where subpopulations learn more or less frequently from their own group compared to the other group. In our simulations, minority–majority-structured populations with moderate degrees of in-group preference better spread and maintained an adaptation compared to populations with more equal-sized groups and weak homophily. Minority groups act as incubators for an adaptation, while majority groups act as reservoirs for an adaptation once it has spread widely. This means that adaptations diffuse throughout populations better when minority groups start out knowing an adaptation, as Indigenous populations often do, while cohesion among majority groups further promotes adaptation diffusion. Our work advances the goal of this theme issue by developing new theoretical insights and demonstrating the utility of cultural evolutionary theory and methods as important tools in the nascent science of culture that climate change adaptation needs.

This article is part of the theme issue ‘Climate change adaptation needs a science of culture’.

## 1. Introduction

Climate change threatens societies worldwide [1], but often most severely affects populations least responsible for greenhouse gas emissions [2]. Help from rich countries most responsible for the emissions is unreliable [3]. To maximize the chances that climate change adaptation efforts succeed, it is critical to understand how basic social factors affect climate change adaptation outcomes. Here, we focus on how the diffusion of adaptations is affected by group structure within a *metapopulation* (i.e. a population of populations or a network characterized by strong *community structure* [4]) and how frequently individuals learn from others within one’s own group versus from individuals from other groups (i.e. *homophily*). Even though anthropogenic global warming is accelerating and intensifying environmental change, local and Indigenous populations often already know of valuable adaptation strategies given that their livelihoods are directly associated with a constantly changing environment, as was the case for their ancestors [5]. Qualitative evidence suggests that existing strategies to promote climate change adaptation are most successful when local stakeholders participate, with community-based adaptation efforts being one important approach to community involvement in climate change adaptation [6]. Despite this evidence, there are open questions as to how adaptive practices spread through heterogeneous populations, which is essential to adaptation success [7]. We are particularly interested in metapopulations characterized by minority–majority structure since this is a characteristic of many subsistence populations living on the economic periphery of more market-integrated populations [8,9]. To understand how adaptive behaviours or practices diffuse through metapopulations, we developed an agent-based model of the spread of an adaptive behaviour or practice to understand when, how and which forms of minority–majority structure promote the diffusion of adaptations. This minority–majority group model setup is the simplest non-trivial metapopulation structure. It represents two groups where one group, the *majority group*, outnumbers the other, the *minority group*, and each group learns more or less frequently from its own group and the other group, specified by group-level homophily.

*Adaptation* has several meanings within climate science, but here we adopt a general definition consistent with anthropological use [10,11] and suitable for studying the spread of culturally learned behaviours [12], including climate change adaptation or mitigation strategies. We define an *adaptation* as a solution to a problem that confers a greater fitness to those who employ the behaviour compared to those who do not [10]. We assume that the adaptation already exists, generated through some adaptive cultural process [13]. *Adaptation success* in our model, therefore, is whether or not the adaptation successfully spreads to all simulated individuals (i.e. *agents*) in the model, and not the innovation of a novel adaptation *per se*. This represents the cases where, for example, an adaptation is introduced by a development actor such as a local government or international development agency [6,9]. It also may represent the case where an adaptation already exists and has been maintained through intergenerational transmission, for example, among Indigenous populations [9,14]. *Adaptation failure* in our model is represented by the loss of the adaptive behaviour from the population, i.e. all agents adopt the non-adaptive behaviour. Note, then, that it is possible for model agents to revert to the non-adaptive behaviour through social learning after learning the adaptive behaviour (the exact model learning process is explained in §2 below). When either adaptive success or failure occur we say that either the adaptive or non-adaptive trait has *fixated*, respectively. We will show that adaptation success is significantly more likely when minority groups start out knowing the adaptive behaviour, indicating their role as adaptation *incubators*. We will show that adaptation success is also significantly more likely when the majority group has a relatively high degree of homophily in order to protect the adaptive behaviour once it has diffused into the majority group, indicating the majority group’s role as an adaptation *reservoir*, preserving the adaptation from cultural extinction. Our work here focuses on adaptation in the context of climate change, but our model and results extend to the broader process of the diffusion of any beneficial, culturally transmitted behaviour in heterogeneous populations.

Our minority–majority group structure is homologous to theoretical core–periphery social structures, where the *core* group is typically larger with most of its connections being in-group connections, while the groups on the *periphery* have smaller populations and have relatively more connections to the core group than *vice versa* [10]. Too often, socially peripheral groups are not included in planning or implementing climate change adaptation efforts, which impedes the diffusion of adaptive practices [7]. Empirical and theoretical work in cultural evolution has shown that homophilous, heterogeneous social structure, characterized by community structure of the networks on which innovations diffuse, promotes greater cumulative cultural complexity [15–17]. Diversity in social structure can support problem-solving [18,19] and prescient ideas often emerge from the peripheries of metapopulations [20]. While homophilous subgroups can promote the development of adaptations and support minority groups, social cohesion can also stifle innovation and lead to conflict [8,21].

We chose to construct our model in terms of *majority groups*, *minority groups* and *homophily levels*, instead of *core* and *periphery* or other potential names, because the meaning is transparent: the minority group is the one with fewer group members compared to the majority group, and group-specific homophily levels modulate how frequently group members learn from others from their own group compared to members of the other group. This choice allows us to understand the effect of semi-structured learning: in our model, teacher selection is not fully constrained by a social network that would assume no social learning occurs outside of one’s social connections. A *teacher* in our model is any agent from whom another agent learns either the adaptive or non-adaptive behaviour. At the same time, our model population is not well-mixed, which would mean social learners choose a teacher independently of group membership. Our work, then, complements related studies that used alternative model formulations. For example, Lieberman *et al.* [22] found that network structure strongly constrained adaptive trait fixation and evolutionary game dynamics. On the other hand, Deffner & Kandler [23] found that idealized agents evolved strategies to balance fast innovation with more sustainable long-term adaptations in a well-mixed, fitness-biased learning model; in that model, simulated learners chose a fully random subset of teachers from a large population, then learned from the best-performing teacher. Our minority–majority approach takes a middle ground, representing the fact that human social networks constrain who we interact with, but we also regularly interact with strangers.

Our model most closely represents those climate change adaptations that may spread from one person or household to another. The case of climate change adaptation in South Pacific Island nations provides several useful examples to which our model may be applied, where the spread of adaptations has been observed to require dedicated inclusion of minority-group populations often spread out among several islands, physically and socially separated from urban, governmental centres located on separate islands [24–26]. Torres Strait Islanders in the South Pacific, for example, have historically dealt with non-anthropogenic climate change, and have culturally evolved practices for tracking seasonal weather patterns and timing crop planting that have not widely diffused to all who might benefit from adopting them [14]. Adaptive practices like this for subsistence farming will soon be widely in demand due to anthropogenic climate change [27]. Such practices tend to diffuse predominantly through person-to-person or household-to-household learning [28,29]. Mangrove ecosystem management is another strategy known by South Pacific Islanders for mitigating sea-level rise that could spread person-to-person [5]. Mangrove ecosystem management is likely a more successful strategy for mitigating rising sea levels compared to seawall construction often promoted by international development agencies. Seawalls often fail because they do a better job keeping water inland once water has breached a wall, effectively acting as a maladaptive dam [30]. Some local and Indigenous South Pacific Islanders know of the benefits of mangrove management and the harms of seawalls, but many others do not. Such maladaptation often occurs when urban-based governments implement plans developed by rich-country development actors and ignore local, Indigenous knowledge [6,9,26]. In general, local, Indigenous residents of a place have historically dealt with non-anthropogenic climate change, and have a repertoire of strategies that could effectively deal with the problem, if only others would adopt them instead of exogenously planned projects [9].

Person-to-person or household-to-household social learning dynamics have been observed in other climate change adaptation cases where adaptation success requires the widespread diffusion of an adaptive practice, such as the adoption of residential rooftop solar photovoltaic installations [31]. Larger-scale climate change adaptation projects that require institution- or government-level change, such as transitioning away from fossil-fuel-burning power generation, may require explicit modelling of those institutions and their constituents [32,33]. Even at the institutional level, the model presented here may provide useful context for understanding knowledge transfer among constituents who help decide which actions their institutions will pursue.

## 2. Model

To understand how minority groups can incubate climate change adaptation and how majority groups can preserve climate change adaptation, we developed an agent-based model to represent a community metapopulation as simulated individuals—*agents*—who perform behaviours with different fitness; agents interact to learn behaviours from other agents [34]. Model metapopulations are composed of two groups: one is the minority group that accounts for a fraction *m* ≤ 0.5 of the total metapopulation, *N*, while the other group is the majority that accounts for a fraction 1 − *m* of the metapopulation.

Following a cultural evolutionary approach, adaptive and non-adaptive behaviours are each represented as a trait held by each agent. We assume that one agent from the minority, one agent from the majority or one agent from each group begins the simulation with the adaptation. Traits are transmitted between agents through payoff-biased social learning [35,36] to give social learning the greatest possible chance of success, i.e. we continue to focus on the ideal case. Social learning is where homophily matters, since homophily specifies to what extent learners prefer teachers from their own group. Group structure and social connectivity are specified via model parameters of homophily and group size. Our primary outcome measure is the success rate, i.e. how frequently adaptation success occurred over 1000 simulation trials. We explain the model dynamics, parameters and computational analyses in more detail below.

To harmonize our presentation with the standard *Overview, Design and Details* (ODD) protocol, we have already introduced the *purpose* (ODD ‘overview’) and *design concepts* (ODD ‘design’) of our model; the *variables* and *process overview* (also from the ODD ‘overview’) and *initialization*, *input* and *submodels* (ODD ‘details’) are described in detail below [37].

### (a) Model dynamics

The model dynamics proceed in three consecutive stages: first, agents are initialized with a group identity, group-level homophily, and whether they practice the adaptation or not. Homophily is represented by the agent’s preference to learn from within their group. Specifically, homophily specifies how much more frequently they learn from their in-group (equation (2.1)) compared to their out-group (equation (2.2)). On each time step, agents select which group to learn from, then select a teacher from the chosen group. Next, the agents engage in one round of learning per time step until one behaviour or the other fixates in the simulated metapopulation, meaning all agents have trait *a*, or all have *A*.

### (b) Initialization

We assume that at *t* = 0 there is an adaptive trait *a* that is introduced into the population by one individual in either the minority group or the majority group, or one individual in each group, while the rest of the population has non-adaptive trait *A*. We assume the fitness of trait *a* is greater than the fitness of trait *A*, written *f*(*a*) > *f*(*A*), where *f*(*T*_*i*_) represents the fitness of agent *i*’s trait *T*_*i*_. Minority and majority group members are initialized with static homophily values *h*_min_ and *h*_maj_, respectively. Homophily can take values continuously between 0 and 1, though we ignore *h*_min_ = *h*_maj_ = 1.0 when the trait is only introduced in one of the two groups since fixation is impossible in this case. When *h*_min_ = *h*_maj_ = 1.0 and both groups are initialized with *a*, then the probability of fixation is the product of the two individual fixation probabilities since the two groups do not learn from one another.

The minority-group fraction, *m*, is set constant to be a fraction of the total population, *N*. In the main text, we set *m* = 0.05 (*m* = 0.20, 0.35, 0.50 tested in the electronic supplementary material) and *N* = 1000 (*N* = 50, 100, 200 tested in the electronic supplementary material). This means that in our simulations analysed in the main text, the minority-group size was 50 and the majority-group size was 950 (table 1).

**Table 1.** Summary of model variables, their meaning and their numerical values used in our computational analyses.

variable	description	values tested (italics = default)
*N*	population size	50, 100, 200, *1000*
*m*	fraction of population in minority group	*0.05*, 0.2, 0.35, 0.5
*h*_min_	minority homophily, specifies in-/out-group learning probability for minority group via equations (2.1) and (2.2)	{0.0, 0.05, 0.1, …, 0.95, 0.99}
*h*_maj_	majority homophily, specifies in-/out-group learning probability for majority group via equations (2.1) and (2.2)	{0.0, 0.05, 0.1, …, 0.95, 0.99}
*A*	non-adaptive, or status quo, behavioural trait	n.a.
*a*	climate change adaptation behavioural trait	n.a.
*f*(*A*)	fitness of non-adaptive behavioural trait *A*	*1.0*
*f*(*a*)	fitness of adaptive behavioural trait *a*	1.05, *1.2*, 1.4, 2.0
*T*_*i*_	behavioural trait of agent *i*	*a*, *A*

### (c) Asymmetric-homophilous learning

At each model time step, each agent selects and learns from another agent, its teacher, weighted by prospective teachers’ group membership and relative fitness within its group. The probability that an agent learns from its own group is

2.1 $$\text{Pr}(\text{Learner chooses in-group teacher})=\frac{1+h}{2},$$

where *h* is the agent’s group’s homophily value. The probability of learning from an out-group member is

2.2 $$\text{Pr}(\text{Learner chooses out-group teacher})=\frac{1-h}{2}.$$

Therefore, the probability with which a learner, *i*, selects a given teacher, *j*, from group *G* is

2.3 $$\text{Pr}(i\text{ selects teacher }j\in G)=\frac{1\pm h}{2}\frac{\;f\;(T_{j})}{\sum_{k\neq i\in G}f\;(T_{k})},$$

where the first fraction in the product on the right hand side of equation (2.3) is the probability of selecting either the in-group ((1 + *h*)/2) or out-group ((1 − *h*)/2), and *T*_*k*_ is the trait of agent *k*. There is no learning noise or miscommunication in this model, so learner *i* adopts its teacher’s trait *T*_*j*_. Trait updating does not occur immediately. First, all agents perform teacher selection and learning, but the learned trait is only adopted after all agents have selected and learned from a teacher, i.e. after the round is complete.

### (d) Stopping condition

The simulation ends with adaptation success or failure, i.e. all agents have trait *a*, or all have *A*.

### (e) Example model dynamics

**Figure 1.**
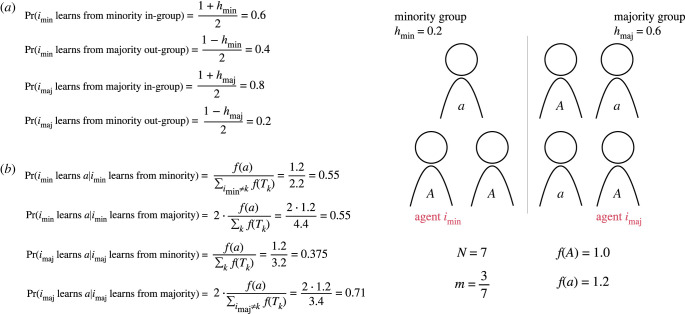
Asymmetric-homophilous learning example. We can break down teacher selection in our model into a two-step process, where first each agent selects which group to learn from, determined by group-level homophily (*a*). Then a teacher is selected at random, weighted by prospective teacher fitness (*b*). The probability that any agent *j* is chosen as a teacher is given by the product of group selection probability and within-group relative fitness of a prospective teacher (equation (2.3)). (Online version in colour.)

To clarify the model, consider the following example learning dynamics for minority- and majority-group members, *i*_min_ and *i*_maj_, respectively, in figure 1. Let the total metapopulation be composed of *N* = 7 individuals and let *m* = 3/7, so three agents are in the minority group and four in the majority. Let the minority have a group-level homophily value of *h*_min_ = 0.2, meaning that minority agents have a 60% chance of selecting a member of their own group to learn from, and a 40% chance of learning from a member of the majority group; let the majority group have a group-level homophily value of *h*_maj_ = 0.6, meaning that a majority-group agent has a 80% chance of selecting a teacher from its own majority group, and a 20% chance of selecting a minority-group teacher (figure 1*a*). Let one agent of three in the minority have the adaptive behavioural trait *a*, and let two members of the four-member majority group have the adaptive behavioural trait. Assume that the non-adaptive fitness is *f*(*A*) = 1.0 and the adaptive fitness is *f*(*a*) = 1.2. Once each agent selects its group, then learning is fitness-biased within the chosen group (figure 1*b*). If *i*_min_ chooses to learn from either its own minority group or the majority group then it has a 0.55 chance of learning adaptive behaviour *a*, since self-learning is not allowed in the model and thus half of the prospective teachers from each group have the adaptive trait, *a*. If *i*_maj_ chooses to learn from the minority there is one agent of three that has the adaptive trait, which results in a probability of 0.375 of learning the adaptive behaviour from the minority group; if *i*_maj_ chooses to learn from its own group, two of the other three agents in its group have the adaptive trait, and so there is a probability of 0.71 that the agent adopts the adaptive behaviour. This process continues for all agents at each time step; the model continues to step until adaptation success or failure, i.e. all agents have trait *a* or *A*, respectively.

### (f) Computational analysis

Our primary outcome variable, the *success rate*, is the frequency of adaptation success across 1000 simulation trials for each parameter setting of interest. We also observed, and calculated the mean of, the number of steps to adaptation success or failure across trials. This will help us understand the time course of the spread of adaptive behaviours, which could be practically useful when evaluating whether or not to abandon an intervention to spread an adaptation.

### (g) Implementation

The model was implemented in the Julia programming language [38] using the Agents.jl package [39]. Plots were made using the ggplot2 library [40] in R [41]. Model and analysis code is publicly available on GitHub (https://github.com/eehh-stanford/SustainableCBA) and the software version used for our Analysis here has a persistent DOI hosted by Zenodo (https://doi.org/10.5281/zenodo.7976114). Simulation output data used for our analysis here is available through the associated Open Science Foundation repository for this project (https://osf.io/cd9hx/).

## 3. Analysis

**Figure 2.**
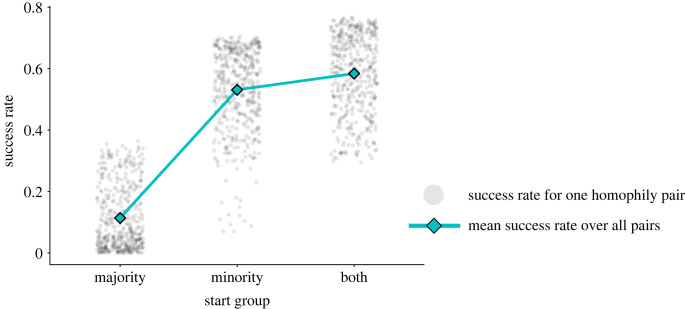
Success rate (*y*-axis) is greater on average when the minority group begins with the adaptation compared to the case where only the majority group begins with the adaptation (‘start group’ on the *x*-axis). Each point represents the success rate from one minority–majority homophily pair, (*h*_min_, *h*_maj_). Not all minority-start-condition success rates are greater than all majority-start-condition success rates. To understand the structure in success rate distributions, we must inspect success rate over specific homophily pairs (figure 3). *N* = 1000, *m* = 0.05 and *f*(*a*) = 1.2. (Online version in colour.)

**Figure 3.**
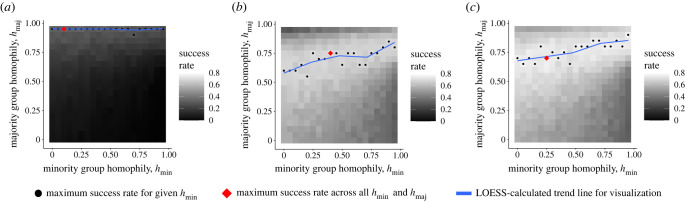
When the minority group starts (*b*) or both groups start with the innovation (*c*), success rates are greater overall compared to when the majority starts with the innovation (*a*), demonstrating the minority incubator effect. Success rates are greater still when both groups start off knowing the adaptive behaviour (*c*). In all three cases, majority group homophily has a greater effect on success rate than the minority-group homophily level, i.e. the majority reservoir effect. *N* = 1000, *m* = 0.05 and *f*(*a*) = 1.2. (Online version in colour.)

**Figure 4.**
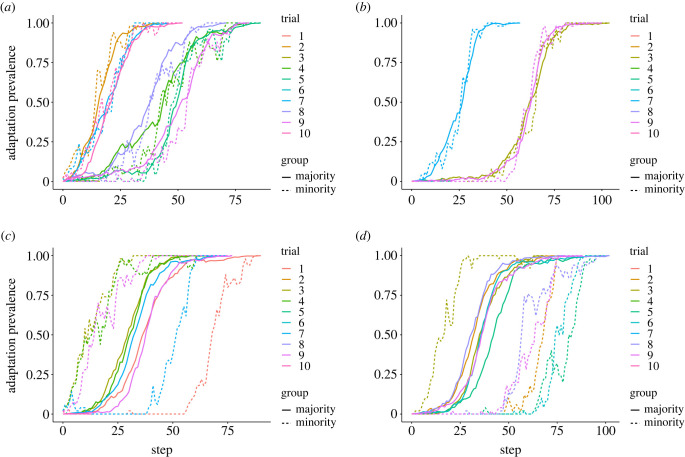
Time series of adaptation prevalence when the adaptation starts in either the minority (*a*,*c*) or in the majority (*b*,*d*) for two symmetric homophily values, *h*_min_ = *h*_maj_ = 0.75 (*a*,*b*) and *h*_min_ = *h*_maj_ = 0.99 (*c*,*d*). Ten trials are shown for all four settings, although many quickly end with adaptation prevalence going to zero. The complementary roles of minority-group incubation and majority-group preservation are exaggerated for extremely high homophily: when the minority group starts with the adaptation, the minority-group members often all learn the adaptation first, incubating the adaptation before it diffuses into the majority group (*c*). When the majority group starts, full adoption in the minority community lags full majority adoption (*d*), which also occurs for some trials as shown in (*c*). For one trial in (*d*), the adaptation diffused into the minority group, where the minority group then acted as an adaptation incubator. (Online version in colour.)

**Figure 5.**
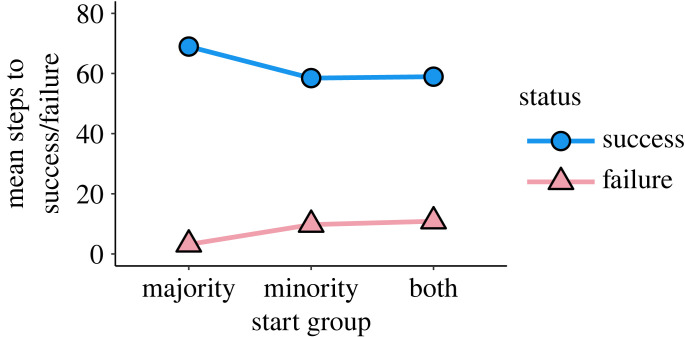
Successful adaptation efforts take significantly longer to achieve on average than failed efforts (mean steps to success or failure on *y*-axis; start group condition on *x*-axis). Success happens faster on average when the minority group starts with the adaptation. *N* = 1000, *m* = 0.05 and *f*(*a*) = 1.2. (Online version in colour.)

**Figure 6.**
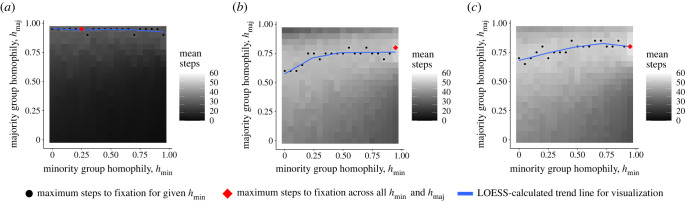
Mean number of steps to fixation across the same group start and asymmetric homophily values tested above. When success rates are greater, the time to fixation increases, indicating that patience is necessary for the spread of adaptations. (*a*) Majority start; (*b*) minority start; and (*c*) both start. (Online version in colour.)

To demonstrate that homophily and group structure can promote adaptation success via minority adaptation incubators and majority adaptation reservoirs, we systematically varied minority and majority homophily levels in the model, *h*_min_ and *h*_maj_, respectively (figure 3), and observed how frequently the adaptive behaviour swept through the population (*success rate*), becoming adopted by each agent. We observed that initializing the adaptation in the minority group is critical to increased success rate (figure 2). However, we also find that success rate is most sensitive to majority homophily whether the adaptation is initialized in the minority group, the majority group or both, which indicates it is important for the majority group to guard its adaptive reservoir in case the adaptation is lost among the minority group through drift (figure 3). To confirm our interpretation that minority groups act as incubators and majority groups as reservoirs, we inspected individual simulation time series and observed some cases where majority adaptation adoption lagged behind minority adoption (minority incubator), and some cases where the majority population had accumulated a large proportion of adopters while the minority adopter prevalence fell or vanished (figure 4). Finally, we analysed the number of time steps to adaptation success or failure across our simulation trial conditions—adaptation success takes longer, while failures ‘fail fast,’ which highlights the need for patience and resources once an adaptation begins to take hold in a minority–majority-structured population (figures 5 and 6).

### (a) Minority-group adaptation-incubator effect

Minorities are critical to better chances of adaptation success, and in fact smaller minorities do better than larger minorities. First, note that overall success rate was significantly lower on average across all *h*_min_ and *h*_maj_ settings when the adaptation was initialized in the majority group (figure 2), and in many settings the success rate is 0 (figure 3). In our sensitivity analyses, we set the minority fraction to *m* = 0.2 and observed maximum success rates of 0.6 (electronic supplementary material, figures S4 and S5, top row), whereas in our main analysis success rates maxed out around 0.7 (figure 3). Maximum success rates were reduced further when *m* = 0.35 and when group sizes were set equal, *m* = 0.5 (electronic supplementary material, figures S4 and S5, middle and bottom rows).

Why should smaller minority group populations improve adaptation success rates, and why should certain values of homophily amplify this effect? First, consider the difference in minority sizes, ignoring the effect of homophily. Consider the initial case where one agent in the minority has the adaptive trait. Smaller minority sizes result in a higher probability of selecting the agent with the adaptive trait at random when an agent must first select which group to learn from, as we have implemented here. In our model, with *N* = 1000 and *m* = 0.05 there is a 1/2 x 1/50 = 1/100 probability that the agent with adaptation is selected at random. When *m* = 0.2 this probability decreases to 1/2 x 1/200 = 1/400. Homophily contributes to this *incubator effect* by leading members of the minority to focus more on what their in-group is doing, and their in-group is the one with the beneficial adaptation. As long as homophily is not too great, the adaptation will diffuse into the majority group as well. When *f*(*a*) = 1.05 ≈ *f*(*A*), adaptations mostly fail to diffuse through the metapopulation at all when they start in the majority group only; when *f*(*a*) = 2.0, adaptations initialized in the minority group always succeed across a wide range of *h*_min_ and *h*_maj_, but often fail when initialized in the majority (electronic supplementary material, figures S7 and S8).

### (b) Majority-group adaptation-reservoir effect

While minority-group participation is essential to incubate an adaptation, we found that majority-group homophily had the largest effect overall on adaptation success. For any value of *h*_maj_, increased *h*_min_ does not change the success rate much, but when *h*_maj_ is set to its optimal value for a given *h*_min_, the success rate roughly doubled (figure 3). This indicates that majority groups have an important role to play as well, namely that of an adaptation reservoir. Once enough majority members learn the adaptive behaviour, the majority group has a greater cultural inertia that will help maintain the adaptation with less adoption variance compared to the minority group, and so can rescue the adaptation from extinction when the adaptation vanishes from the minority group.

### (c) Time series of adaptation diffusion support this interpretation

If minority groups do indeed act as incubators, and majority groups as adaptation reservoirs, then this should be reflected in the time series of adaptation prevalence in the two groups. Indeed, time series of adaptation prevalence among the two groups further supports the interpretation that the groups have complimentary incubator-reservoir roles (figure 4). For approximately optimal homophily levels *h*_min_ = *h*_maj_ = 0.75, identified by reading off the heat maps in figure 3, we see some cases where adaptation success was preceded by minority incubation when the minority starts with the innovation (figure 4*a*). However, even when the minority group starts with the innovation, some adaptation successes depended on the majority group protecting the adaptation while the adaptation vanished from the minority group. Similarly, when the majority started with the adaptation, we see cases where the majority again protects the relatively rare adaptation before adaptation success (figure 4*b*). However, in this same setting there is one trial where the adaptation diffused into the minority group after starting in the majority group, and the minority group incubated the adaptation for a period.

#### (i) Successful adaptation takes time

To complete our analysis, we calculated the mean time to achieve adaptation success or failure across each group-start condition broken out by success or failure. We also calculated the mean time to fixation across successful and failed adaptation efforts across all homophily settings. Success was achieved faster when the minority group or both groups started with the adaptation (figure 5). The region of maximal time steps to fixation mostly mirrors the region of maximal success rate in the heatmaps in figure 3. This indicates that patience is required for successful adaptations. It also suggests that failure will be relatively quick. This makes sense, since many more agents will have to adopt the adaptive trait for adaptation success, while relatively few with the adaptive trait will need to adopt the non-adaptive trait at the beginning of the simulations. Homophily can also affect the time to fixation, with higher minority-group homophily resulting in longer times to fixation (figure 6*b*,*c*), but no boost in the success rate. In the electronic supplementary material, we examine how different parameter settings for the population size, *N*, minority-group fraction, *m* and adaptive behaviour fitness, *f*(*a*) affect the number of time steps to success or failure. Briefly, time to fixation is inversely correlated with *N* (electronic supplementary material, figure S3); time to fixation is relatively unchanged by changes to *m* (electronic supplementary material, figure S6); and time to fixation is inversely correlated with *f*(*a*) (electronic supplementary material, figure S9).

## 4. Discussion

In this paper’s idealized simulations, relatively small minority groups served an essential role as adaptation incubators, while homophilous majority groups supported the diffusion of adaptations by acting as an adaptation reservoir, with more agents available to maintain the adaptation than the smaller minority population. Therefore, it is practically important to include minority groups in adaptation efforts, as well as being the equitable, just, morally upstanding thing to do. Adaptation success took significantly longer than failure, so patience and persistence are required, even in the ideal case. Our approach to understanding minority–majority dynamics used mechanistic modelling of cultural evolution, which should continue to serve an important role to connect individual- and dyadic-level cognitive learning mechanisms with more complex, but possibly less concrete, models of climate change adaptation dynamics [42]. In general, mechanistic, agent-based modelling approaches such as ours help social scientists avoid sprawling verbal theories that may be mismatched to statistical models that are unsuitable for causal inference [43–45]. Moreover, stochastic agent-based models enable the inspection of path dependence on social outcomes [46,47], including non-equilibrium social dynamics that other approaches may not generate [48]. Still, alternative formal approaches to modelling the diffusion of adaptations in minority–majority metapopulations could provide complementary insights—for example, a population-genetics approach might explain that minority groups act as better incubators compared to majority groups because selection is weaker when the adaptation is more rare [49, ch. 3].

We assumed that all adaptations are identically transmissible, but cognitive, cultural and physical constraints are known to be important for predicting the cultural spread of information [50,51]. For example, just as our physical bodies constrain the sort of cultural information that humans generate and transmit between individuals in the laboratory [52], some adaptive traits may be favoured due to shared in-group cultural experiences, which could be helpful for amplifying climate change adaptation [53]. Complex or taboo adaptive behaviours may require multiple teaching exposures before an individual adopts them [54], but we assumed that a single exposure was always sufficient—modifying this single-exposure assumption may result in lower success rates. Inter-group enmity and discrimination, which we ignored in this model, could further undermine adaptation success [55]. Minority and majority groups may also influence culture in different ways, with minority influence possibly exerting influence indirectly but persistently [56,57], which may boost success rates by strengthening the minority-group adaptation-incubator effect. Furthermore, we assumed that there is just one pre-existing trait that determines adaptive fitness. In reality, fitness is based on a suite of cultural traits that are often correlated, both in their expression and their transmission [58,59]. Furthermore, different traits or behaviours are often composed to form new composite cultural variants through cumulative cultural evolution [60,61]. Group structure is known to co-evolve with cumulative cultural traits [15,62,63], which could have complex, unpredictable effects on adaptation success rates. Finally, we assumed that both the minority-group and majority-group members received the same fitness boost by adopting the adaptive behaviour. In reality, however, an adaptation is likely to provide different value to different stakeholders. For example, mangrove planting and management may help mitigate sea-level rise along the coast in South Pacific Island nations [5], but it does not directly help subsistence farmers deal with changing weather patterns in the highlands of these nations.

The principle of ‘fail fast’ is well-known to software developers who move quickly and break small things as they build big things. Fail fast has also been identified as an important strategy for organizations [64]. This suggests that international development actors, local governments and citizens implementing climate change adaptations should plan for a few quick failures, with adjustments in between trials, before the adaptation gains the sort of critical momentum to spread through the population. Furthermore, since adaptation success takes significantly longer than failure, planners should also account for extended periods of financial and technical support as adaptations spread. Pisor *et al.* [9] suggest that social insurance like basic income could facilitate climate change adaptation in Indigenous and other subsistence populations by cushioning the downside risk of fast failure of potential climate innovations. Our model did not include any mechanism for learning from past failures, although learning from past adaptation failures has been identified as an important step for successful community-based (and other) adaptation efforts [65].

Our results support the suggestions by Pisor *et al.* [9] and Jones *et al.* [10] that subsistence, frequently Indigenous, populations on the margins of larger more market-integrated populations might be a source of climate adaptation. Moreover, our results support the hypothesis that successful innovations tend to emerge from the peripheries of networks [66], rather than in the cores of networks [67]. These observations suggest the potential functional importance of minority communities for innovation and adaptation. As such, it is essential that minority populations retain cultural autonomy [9]. Hegemonic cultural forces can easily homogenize diverse populations. Models by Bunce & McElreath [8] suggest potential means, through the construction of protected ‘homelands’ and resulting asymmetric interactions, by which minority cultural norms can be retained, even when there is a strong tendency for homogenization. In this regard, our results on the efficacy of minority-group-initiated adaptations, the results of Bunce & McElreath [8] on retention of minority norms, and the results of Derex & Boyd [15] on community structure in transmission networks facilitating greater cumulative cultural evolution, seem to be converging on a robust pattern: in the ideal case, population heterogeneity in the form of group structure tends to promote the diffusion of adaptive behaviours and practices.

## Data Availability

Our simulation output data and code used to generate our analysis figures is available from the Dryad Digital Repository: https://doi.org/10.5061/dryad.2bvq83bwm [68]. This is part of the Climate Change Adaptation Needs a Science of Culture data portal from the Dryad Digital Repository: https://doi.org/10.5061/dryad.bnzs7h4h4 [69]. Our model code used to generate our analysis is also available from the GitHub repository: https://github.com/eehh-stanford/MinMaj-Adaptation-Diffusion [70]. Additional analyses are provided as electronic supplementary material [71].
